# Clinicopathological characteristics, diagnosis, and prognosis of pregnancy‐associated breast cancer

**DOI:** 10.1111/1759-7714.13045

**Published:** 2019-03-28

**Authors:** Bin Wang, Yanfang Yang, Zhansheng Jiang, Jing Zhao, Yiran Mao, Jun Liu, Jin Zhang

**Affiliations:** ^1^ Key Laboratory of Cancer, Prevention and Therapy National Clinical Research Center for Cancer, Tianjin Medical University Cancer Institute & Hospital Tianjin China; ^2^ Key Laboratory of Breast Cancer Prevention and Therapy Tianjin's Clinical Research Center for Cancer, Tianjin Medical University, Ministry of Education Tianjin China; ^3^ Second Department of Breast Cancer; ^4^ Department of Integrative Oncology; ^5^ Department of Ultrasound Diagnosis & Treatment; ^6^ Third Department of Breast Cancer

**Keywords:** Clinical characteristic, pregnancy‐associated breast cancer, prognosis, therapeutic strategy

## Abstract

**Background:**

The aim of this study was to evaluate the characteristics, diagnosis, prognosis, and effective treatment modalities of pregnancy‐associated breast cancer (PABC).

**Methods:**

From 1 January 2005 to 31 December 2015, 142 patients with PABC were identified in the Cancer Institute and Hospital of Tianjin Medical University database. The clinicopathological features, treatment methods, and outcomes were retrospectively evaluated.

**Results:**

The median age at diagnosis was 30 years. All patients presented with a palpable mass in the breast. The sensitivity of ultrasound and mammography in PABC diagnosis was 86% and 83.3%, respectively, which increased to 91.3% when a combination of mammography and ultrasound was used. The median tumor size was 5.5 cm, and 63.1% of patients had associated axillary lymph node metastases. The proportions of ER negative, PR negative and HER2 positive were 45.7%, 45.7% and 30%, respectively. The five‐year overall survival (OS) and disease‐free survival (DFS) rates were 76.8% and 63.5%, respectively. According to univariate analysis, T stage, N stage, and HER2 status were significant prognostic factors for OS and DFS. The time interval between the onset of the first symptom and the first meeting with a health professional was also significant for OS. Multivariate analysis showed that T stage and HER2 status were independent prognostic risk factors for OS and DFS.

**Conclusion:**

PABC is an aggressive form of breast cancer associated with advanced stage at diagnosis. Despite the existing difficulties in diagnosis, imaging examinations are indispensable. Early diagnosis and multidisciplinary therapy, including anti‐HER2 targeted therapy, may be important to improve prognosis.

## Introduction

Pregnancy‐associated breast cancer (PABC) is commonly defined as breast cancer diagnosed during pregnancy or within 12 months following delivery. It is a rare and peculiar type, accounting for 0.2–3.8% of all breast cancers,^1^ but its incidence in women aged < 45 years varies from 2.6% to 7%,^2^, ^3^ and rises to 15.6% in women aged > 35 years.^4^

PABC is generally recognized as a particularly aggressive type of cancer for several reasons: young age at diagnosis, advanced T stage, high rate of lymph node involvement, negative estrogen ER and PR status, and high rate of HER2 overexpression. Previous studies have yielded controversial results regarding PABC prognosis. Most studies describe it has having adverse biological characteristics with a relatively poor outcome,^5^, ^6^, ^7^, ^8^ while other studies have suggested that the poor survival rates are the result of adverse pathologic features rather than pregnancy.^9^, ^10^

In this retrospective study we reviewed the clinical characteristics, imaging, and pathologic features of 142 PABC patients who had been diagnosed and treated in our hospital to evaluate the therapeutic strategies and analyze prognostic factors.

## Methods

### Study population

Among the 40 000 patients with breast cancer who were admitted to the Cancer Institute and Hospital of Tianjin Medical University from 1 January 2005 to 31 December 2015, 142 (0.36%) were diagnosed with PABC. Institutional Review Board approval was obtained for this study. Medical records, including patient and tumor characteristics, and diagnosis and treatment modalities, were retrospectively reviewed.

Clinical staging was based on the tumor node metastasis (TNM) staging of breast cancer developed by the American Joint Committee on Cancer. Immunohistochemical analysis of ER, PR, and HER2 was performed in our pathology department using formalin‐fixed, paraffin‐embedded tissues. The primary antibodies used in this study were: ER (SP1, 1:200 dilution), PR (SP2, 1:200 dilution), and HER2 (CB11, 1:600 dilution; Zymed, San Francisco, CA, USA). The threshold for ER and PR positivity was 1%. Regarding HER2/neu assessment, a standard 0–3+ scoring system based on membrane staining by immunohistochemistry was used. Intensity patterns with scores 0–1+ were considered negative, while 3+ was considered positive; those scored as 2+ were further assessed by fluorescence in situ hybridization.

#### Follow‐up

Clinical follow up was performed by telephone in all patients. At the follow‐up deadline on 30 July 2018, 140 patients with PABC had completed the follow‐up. Two cases, at clinical stages II and III, were lost to follow‐up after one cycle of neoadjuvant chemotherapy. Disease‐free survival (DFS) was defined as the interval from PABC pathological diagnosis to the date of relapse or metastasis. Overall survival (OS) was defined as the interval between PABC pathological diagnosis and the date of death as a result of the disease or the last follow‐up.

#### Statistical analysis

Data management and statistical analysis were performed using SPSS version 20.0. The χ^2^ test was used to assess categorical variables and the Student's independent *t*‐test to compare continuous variables. Kaplan–Meier curves and log‐rank tests were used to assess survival outcomes and single‐factor analysis. Multifactor survival analysis was performed using the Cox risk ratio model. *P* < 0.05 was considered statistically significant.

## Results

### Clinical characteristics

The clinical characteristics of the patients are shown in Table 1. The median and mean ages were 30 and 30.3 years (range: 24–44 years), respectively. Fifty of the 142 (35.2%) patients had a family history of malignancy, including 21 (14.8%) with a history of breast cancer. A total of 112 (78.9%) patients were diagnosed during lactation and in the remaining 30 patients (21.1%) breast cancer was discovered during pregnancy, including 12 cases in the first trimester, 14 cases in the second trimester, and 4 cases in the late trimester of pregnancy. Within the antepartum subgroup (*n* = 30), three patients in the first and two patients in the second trimester of pregnancy voluntarily chose to terminate the pregnancy. One patient in the second trimester had a preterm delivery.

**Table 1:** Clinicopathological features of 142 PABC patients

Variables	No. of patients (%)
Patients	142
Age in years, median (range)	30 (24–44)
Onset period	
First trimester	12 (8.4)
Second trimester	14 (9.9)
Late trimester	4 (2.8)
Postpartum	112 (78.9)
Family tumor history	
Yes	50 (35.2)
No	92 (64.8)
Initial symptom	
Mass	130 (91.5)
Mass and nipple discharge	12 (8.5)
First pregnancy or delivery	
Yes	70 (49.3)
No	72 (50.7)
Age of first delivery, median (range)	27 (21 ~ 39)
Breastfeeding	
Yes	130 (91.5)
No	12 (8.5)
Breast involved	
Right	82 (57.7)
Left	60 (42.3)
Tumor staging	
T_1_	8 (5.6)
T_2_	68 (47.9)
T_3_	38 (26.8)
T_4_	20 (14.1)
Unknown†	8 (5.6)
Axillary lymph node metastasis	
Positive	86 (60.6)
Negative	56 (39.4)
Clinical staging	
I	8 (5.6)
II	64 (45.1)
III	60 (43.3)
IV	10 (7.0)
Pathological type	
Invasive ductal carcinoma	130 (91.5)
Invasive lobular carcinoma	2 (1.4)
Other types	10 (7.1)
Lymph node metastasis‡	
0	48 (36.9)
1–3	30 (23.1)
≥ 4	52 (40)
Histological grading§	
II	54 (41.5)
III	22 (17.0)
Unknown	54 (41.5)
Immunohistochemical markers¶	
ER positive	76 (54.3)
PR positive	76 (54.3)
HER2 overexpression	42 (30)
KI67 positive††,‡‡	114 (89.1)
P53 positive††	82 (68.3)
Molecular subtype¶	
Luminal A	10 (7.1)
Luminal B	66 (47.1)
HER2 overexpression	32 (22.9)
TNBC	32 (22.9)

† Patients underwent excision biopsy at local hospitals. Data available for:

‡ 130 cases;

§ 76 cases;

¶ 140 cases; and

†† 120 cases.

‡‡ KI67 ≥ 14% was considered positive.

PABC, pregnancy‐associated breast cancer; TNBC, triple negative breast cancer.

All patients self‐discovered a breast mass as the initial symptom: nine patients showed red and orange peeling skin, six had nipple discharge, four had papillary hemorrhagic discharge, and two had purulent discharge. The mean PABC size was 5.5 cm (range: 2–16 cm) and clinical nodal involvement at diagnosis was observed in 60.6% of patients. The clinical PABC stage was advanced: only 5.6% of patients were at stage I, while stages III and IV accounted for 35.2% and 15.5% of all patients, respectively.

### Pathological features

The most common histologic PABC type was invasive ductal carcinoma, accounting for 91.5% (130/142), followed by medullary carcinoma (*n* = 4), invasive lobular carcinoma (*n* = 3), mixed mucinous carcinoma (*n* = 1), tubular carcinoma (*n* = 1), and squamous cell carcinoma (*n* = 1). Histological grading was available for 76 patients, consisting of 54 cases in grade II and 22 cases in grade III. Eighty‐two cases showed lymph node metastasis: 30 cases with 1–3 lymph node metastases and 52 cases with > 3 lymph node metastases. A total of 140 patients underwent ER, PR, and HER2 examination, and the positive expression rates were 54.3%, 54.3%, and 30%, respectively. Ki‐67 examination was available for 120 patients and the positive rate was 89.1% according to the limit of 14%. Based on the immunohistochemistry results, the four molecular subtypes were: Luminal A (*n* = 10), Luminal B (*n* = 66), HER2 overexpression (*n* = 32), and triple‐negative breast cancer (*n* = 32). The pathological features are shown in Table 1.

### Preoperative diagnosis and treatment

Oncological management and treatment modalities are summarized in Table 2. The average delay in time to see a doctor and make a definite diagnosis were 6.07 months (range: 1 day–40 months) and 7.84 months (range: 3 days–100 months), respectively. Of the 30 women in the antepartum subgroup, five patients voluntarily chose to terminate the pregnancy to undergo further treatment, while 25 women postponed medical advice until after their delivery. Difficulty in PABC diagnosis was another reason for delayed diagnosis: 24 patients were misdiagnosed with acute mastitis (*n* = 14), a benign mass (*n* = 4), and milk production (*n* = 6).

**Table 2:** Oncological management of the 142 PABC patients

Variables	No. of patients (%)
Average delay to diagnosis (range)	7.84 months (3 days–100 months)
Average delay to see a doctor (range)	6.07 months (1 day–40 months)
False differential diagnosis†	
Acute mastitis	14 (58.3)
Benign mass	4 (16.7)
Milk production	6 (25)
Type of ultrasound‡	
Cancer	110 (86.0)
Milk production with inflammation	12 (9.4)
Benign mass	3 (2.3)
No diagnosis could be made	3 (2.3)
Type of mammography§	
Cancer	40 (83.3)
Benign mass	4 (8.3)
Mammary dysplasia	2 (4.2)
No diagnosis could be made	2 (4.2)
Ultrasound combined with mammography¶	
Cancer	42 (91.3)
Benign mass	4 (8.7)
Neoadjuvant chemotherapy	
Yes	78 (54.9)
No	64 (45.1)
Type of surgery	
Modified radical mastectomy	70 (49.3)
Conventional radical mastectomy	28 (19.7)
Breast‐conserving surgery	32 (22.5)
Non	12 (8.5)
Adjuvant chemotherapy††	
Yes	126 (96.9)
No	4 (3.1)
Adjuvant radiotherapy	
Yes	78 (54.9)
No	64 (45.1)
Endocrine therapy‡‡	
Yes	64 (84.2)
No	12 (15.8)
Trastuzumab§§	
Yes	24 (57.1)
No	18 (42.9)

† 24 patients were misdiagnosed. Data available for:

‡ 128 cases;

§ 48 cases;

¶ 46 cases;

†† 130 cases who underwent adjuvant chemotherapy;

‡‡ 76 cases with positive ER or PR;

§§ 42 cases with HER2 overexpression.

PABC, pregnancy‐associated breast cancer.

In our study, 128 patients underwent ultrasound examination: 110 (86%) patients had malignant tumors, 12 were assessed with milk production combined with inflammation, 3 as benign mass and 3 could not be diagnosed. Furthermore, 48/142 (33.8%) patients underwent mammography and among them 40 (83.3%) cases resulted in a correct diagnosis, 4 were considered benign masses, 2 were misdiagnosed as mammary dysplasia, and 2 cases could not be diagnosed. The sensitivities of ultrasound and mammography were 86% and 83.3%, respectively. Forty‐six patients in our series agreed to simultaneously undergo the two imaging examinations and 42 patients had correct diagnostic findings or suspected malignancy. The sensitivity of the combination of mammography and ultrasound increased to 91.3%. All PABC cases were eventually diagnosed by large‐core needle biopsy (88 cases) or frozen section biopsy (54 cases).

As most of the PABC cases in our group were at advanced clinical stage, 78 (54.9%) postpartum patients were treated with neoadjuvant chemotherapy. The response to neoadjuvant chemotherapy according to the pathological report was: 22 cases in III degree, 36 cases in II degree, 6 cases in I degree, and 14 cases with no clear response to chemotherapy. We could not evaluate the effect of chemotherapeutics on pregnancy or the fetus, as all 78 patients were postpartum. The only premature baby is currently in good health.

The majority of PABC patients (130/142) underwent surgery, except two patients who were lost to follow‐up and those with metastases at diagnosis. Seventy patients underwent modified radical mastectomy, 28 underwent conventional radical mastectomy, and 32 (22.5%) underwent breast‐conserving surgery (BCS). In our sample, 96.9% of patients were administered adjuvant chemotherapy and 78 were administered adjuvant radiotherapy, including 32 patients who underwent BCS. Sixty‐four patients were administered endocrine therapy, six of whom used goserelin for ovarian castration. Among the 42 patients with HER2 overexpression, trastuzumab (Roche, Basel, Switzerland) targeted therapy was administered in 24 cases.

### Survival analysis

**Figure 1:**
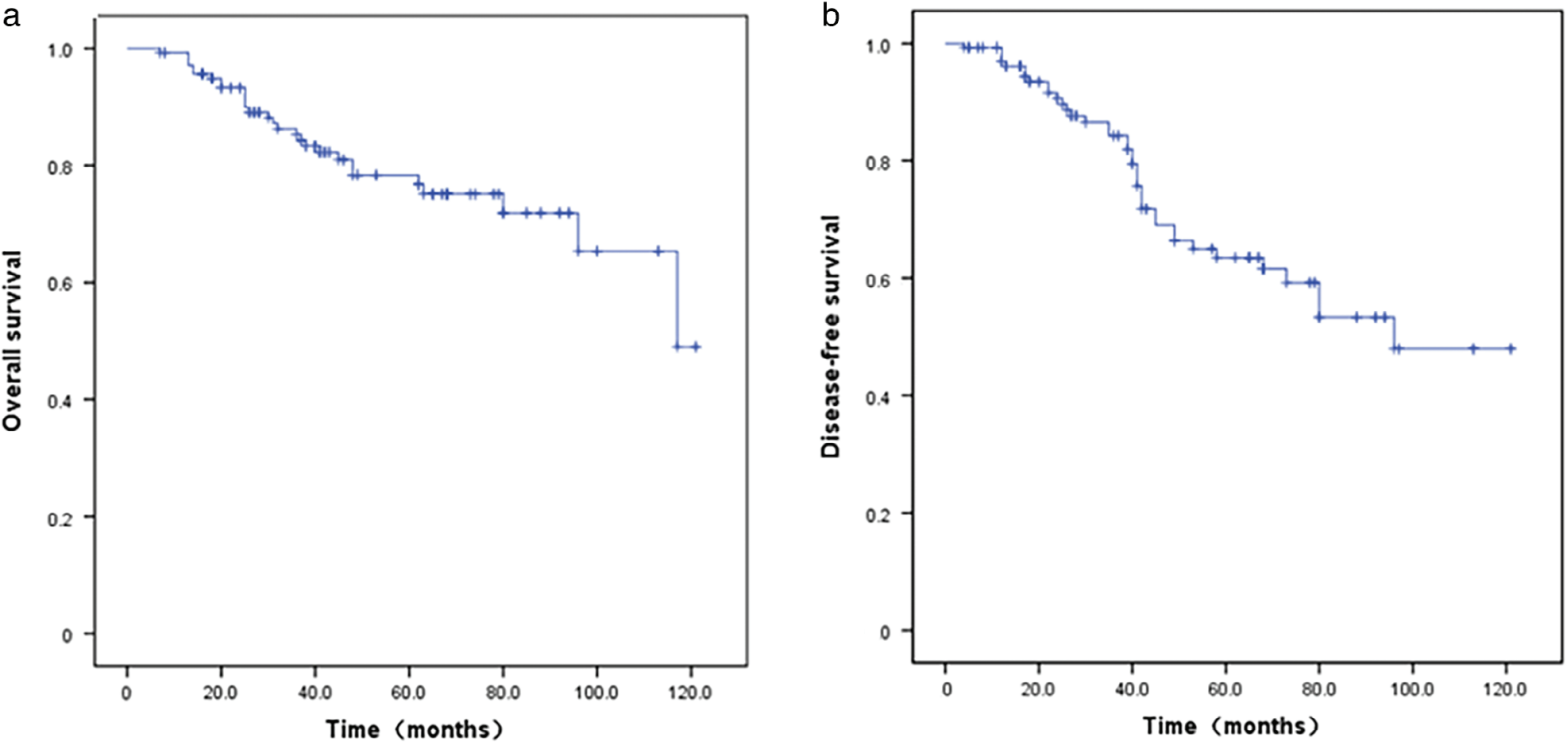
Five‐year (**a**) overall survival (OS) and (**b**) disease‐free survival (DFS) curves of 140 patients with pregnancy‐associated breast cancer (PABC).

During the median follow‐up duration of 63 months, 38 of 140 (27.1%) patients developed local tumor recurrence or distant metastasis and 30 (21.4%) patients died of breast cancer. Among the PABC cases with metastasis, lung metastasis was the most common site, accounting for 61.1% (22/36), followed by bone metastasis (6/36), liver metastasis (5/36), brain metastasis (2/36), and multiple lymph node metastasis (1/36). Univariate analysis using Kaplan–the Meier method showed five‐year DFS and OS rates of 56.1% and 72.3%, respectively. The Kaplan–Meier curves for DFS and OS are shown in Figure 1.

According to univariate analysis, T stage (T1, T2 vs. T3, T4, *P* = 0.048), N stage (N0 vs. N1, N2, *P* = 0.016), and HER2 status (*P =* 0.032) were significant predictors of OS and DFS (all *P* < 0.05). The duration between the onset of an initial symptom and the first meeting with a health professional (*P* = 0.02) was also significantly correlated with OS. Multivariate analysis showed that T stage and HER2 status were independent prognostic factors for DFS and OS (*P* < 0.05) (Table 3).

**Table 3:** Multivariate analysis of prognostic factors affecting OS and DFS of 140 PABC patients

	OS					DFS				
Variables	B	SE	*P*	HR	95% CI	B	SE	*P*	HR	95% CI
TI	−2.688	0.846	0.111	0.068	0.013–0.357					
HER2 status	4.552	1.196	0.000*	14.832	9.089–19.501	2.485	0.938	0.008*	11.997	1.909–15.374
T stage	−2.688	0.846	0.001*	0.068	0.013–0.357	0.753	0.552	0.043*	2.124	0.719–6.270
N stage	−1.602	1.272	0.208	0.202	0.017–2.438	0.152	0.420	0.717	1.164	0.511–2.653

* *P* < 0.05. TI, interval between the onset of the initial symptom and the first meeting with a health professional. CI, confidence interval; HR, hazard ratio; PABC, pregnancy‐associated breast cancer; SE, standard error.

**Figure 2:**
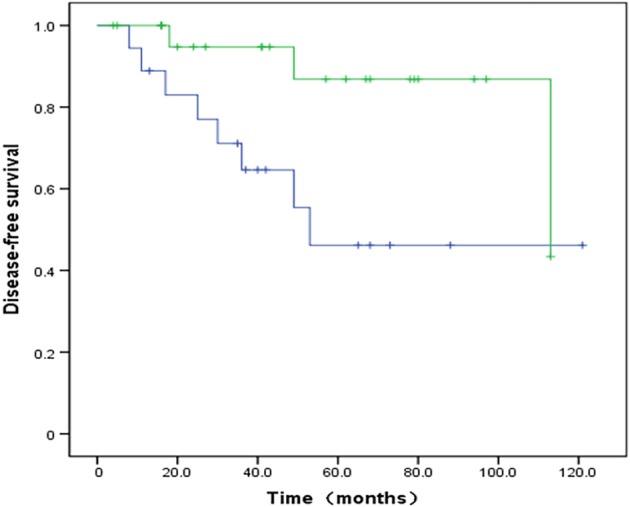
Kaplan–Meier curves showing disease‐free survival (DFS) according to use of trastuzumab targeted therapy. The DFS rate is significantly higher in the trastuzumab therapy group (*P* < 0.01). Non‐trastuzumab group, trastuzumab group. 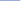 non‐Herceptin group, 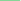 Herceptin group

In the subgroup analysis, patients were stratified as HER2 positive and HER2 negative. PABC cases with HER2 overexpression gained a significant survival benefit from trastuzumab targeted therapy, with significantly improved five‐year DFS (55.6% vs. 87.5% with trastuzumab; *P* = 0.043) (Fig 2).

## Discussion

PABC is a peculiar type of breast cancer with a low incidence. In our study, PABC cases accounted for 0.36% of all breast cancer cases, which is consistent with the reported incidence rate of 0.2–3.8%. Because of the low rate of PABC incidence, no randomized or prospective studies have been conducted regarding optimal diagnostic evaluation, management, and prognosis. In this study, we retrospectively and retroactively analyzed 142 PABC patients to better identify the biologic characteristics.

According to the literature available, the average age of PABC patients varies from 32 to 38 years.^6^, ^11^ In our population, the median and mean onset age was 30 years, slightly younger than the previous reported age, which might be associated with the younger age of onset of breast cancer and obstetrical history in China. In our sample, 14.8% of patients had a family history of breast cancer. Several studies have suggested that a family history of breast cancer should be considered when assessing the risk of PABC.^12^, ^13^

PABC is usually associated with palpable masses in advanced T stage.^14^, ^15^ In our study, all patients presented with palpable masses and 8.5% with nipple discharge at the same time. The mean tumor size was 5.5 cm and clinical nodal involvement was observed in 60.6% of cases, including 4.2% in N3. Several studies have reported that the delay in PABC diagnosis is attributed to its advanced clinical stage.^13^ The average delay to obtain a definite diagnosis was 7.84 months in our cohort, which was consistent with the reported range of 1–13 months.^16^ In addition, delayed diagnosis was one of the negative factors that affected OS in our study. Several reasons, as consequences of pregnancy and lactation, might be associated with the delayed PABC diagnosis, such as patients or doctors overlooking clinical PABC symptoms and difficult radiographic evaluation as a result of changes in breast density. However, postponing medical advice until after delivery was the most prominent reason for a delayed PABC diagnosis in the present study.

**Figure 3:**
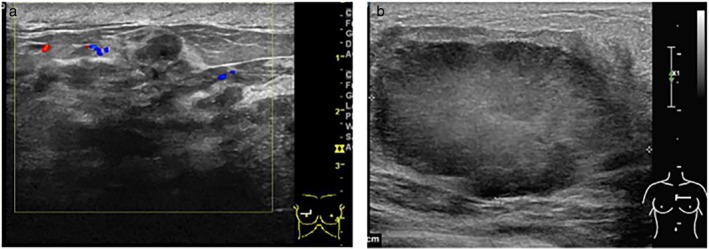
(**a**) Ultrasound shows the structure of the mammary gland disorder, skin thickening subcutaneous tissue space edema, and low echo area, suggesting breast cancer (Grade III invasive ductal carcinoma). (**b**) Ultrasound shows a 5.0 × 2.9 × 4.8 cm hypoechoic, undersmooth, irregular, and lobulated mass, which indicates lobular neoplasms (Grade II invasive ductal carcinoma).

**Figure 4:**
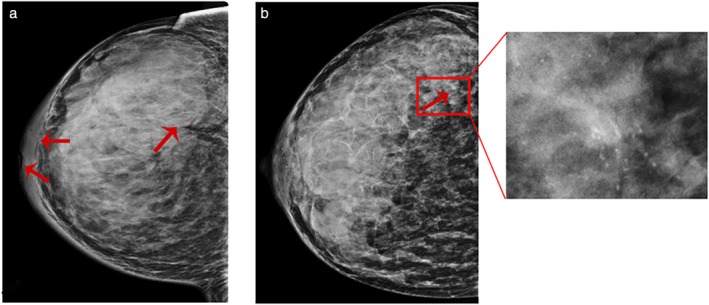
(**a**) Mammography shows isodense masses of irregular shape but mostly smooth margins and crater nipples, as well as pachyderma around the mammary areola, which suggests a malignant tumor (Grade III invasive ductal carcinoma). (**b**). Conventional mammography image displays the high‐density breast with no obvious malignant signs. Architectural distortion and irregular microcalcifications are only shown in magnified mammograms (Grade II invasive ductal carcinoma).

The imaging procedures for PABC diagnosis and staging in our group included both breast ultrasound and mammography. Ultrasound should be performed as first‐rank procedure, as it has almost 100% sensitivity for PABC diagnosis.^17^ However, the accuracy rate of ultrasound examination is lower (86% in our study) (Fig 3a) and also often reveals tumors with regular borders, posterior acoustic enhancement, and parallel orientation, highlighting benign masses (Fig 3b). Our results are consistent with findings by Ayyappan *et al.* who reported that PABC usually presents a falsely reassuring appearance on ultrasound.^18^ Although mammography has been demonstrated to be a safe procedure for pregnant women as irradiation to the fetus is negligible, mammography is thought to be unsuitable for PABC because of high breast density.^19^ Therefore, a very low rate of only 33.8% of patients in our study underwent mammography examination. However, mammography helped to obtain a correct diagnosis in 83.3% of patients with PABC (Fig 4a). Some patients were misdiagnosed as having benign masses or no evidence of abnormality (Fig 4b). Encouragingly, the combined use of mammography and ultrasound increased the accuracy rate to 91.3%. Therefore, both ultrasound and mammography are recommended for pregnant or lactating women because of the risk of delay and difficulties in making a PABC diagnosis. However, contrast‐enhanced breast magnetic resonance imaging (MRI) is not currently recommended for PABC patients considering the unhealthy impact of contrast media on fetal safety.^20^

In our study, invasive ductal carcinoma was the most common pathological type accounting for 91.5% of all patients. In addition, 41.5% of cases were grade II and 17.0% of cases grade III, which was consistent with the results of previous studies showing that PABC tends to be associated with high histological grades.^21^ PABC is thought to exhibit other adverse biologic features, such as more frequent ER and PR negative status, and a high rate of HER2/neu overexpression or amplification.^6^, ^11^, ^22^ In this study, 45.7% of PABC patients were ER and PR negative and 30% showed HER2 overexpression. Higher Ki‐67 (89.1%) and p53 positive (68.3%) levels were also observed. Our results suggest that PABC could be characterized by more adverse tumor characteristics. HER2 overexpression (22.9%) and triple‐negative breast cancer (22.9%) were the more common molecular subtypes, which is consistent with the findings of several other reports.^7^

PABC requires multidisciplinary comprehensive treatment, including surgery, chemotherapy, radiotherapy, endocrine therapy, and trastuzumab targeted therapy. Whether pregnancy termination can improve PABC prognosis remains controversial.^23^, ^24^ In our study cohort, five patients chose to terminate their pregnancy, but did not showing any significant improvement in survival. Surgery is the most important treatment to combat PABC, and is safe during all trimesters of pregnancy.^20^ In our patient cohort, the majority of patients underwent surgery and most (55.7%) were subjected to modified radical mastectomy, likely as a result of the advanced clinical stage. However, no significantly improved survival was observed in PABC patients who underwent surgery. Radiotherapy is not a preferred method to combat PABC, because of the limited available data regarding its effect on the fetus; therefore, it is preferable to postpone radiotherapy until after birth.^15^, ^25^ In our population, 78 postpartum patients were administered radiation therapy, including 34 cases who received BCS; however, there was no clear evidence to indicate that such treatment could significantly improve PABC prognosis.

The use of chemotherapy in PABC patients follows standard recommendations, as in a non‐PABC setting. However, some specific issues should be considered. Among our patients, 96.9% were administered chemotherapy, including 54.9% administered neoadjuvant chemotherapy as a result of advanced stage or poor prognosis. Neoadjuvant chemotherapy was not beneficial to PABC patient survival in our study, but it improved the rate of BCS (22.5%) in PABC compared to approximately 10% in non‐PABC patients in China.^26^ As all patients who received chemotherapy were in postpartum, we could not evaluate the effect of chemotherapeutics on the pregnancy and fetus, but most of the previous literature has shown that chemotherapy can be safely used during the second and third trimesters.^15^, ^20^ The role of endocrine therapy is contraindicated and unclear.^27^ In our study, endocrine therapy was available to 84.2% of ER/PR positive postpartum patients, but statistical analysis did not reveal a survival benefit.

Anti‐HER2 therapy is important for PABC, as patients often present with HER2 overexpression.^28^ Trastuzumab is approved for the treatment of HER2‐positive breast cancer in the neoadjuvant, adjuvant, and metastatic settings. Trastuzumab targeted therapy was administered to 57.1% of HER2‐positive patients, and subgroup analysis showed that it significantly improved the prognosis of HER2‐positive PABC patients. However, a previous study including 34 patients during pregnancy reported that trastuzumab exposure in pregnant patients was associated with pregnancy complications or fetal malformation.^29^ Therefore, it is recommended that trastuzumab targeted therapy is postponed until after delivery.^28^

Although several previous studies have reported that PABC prognosis is similar to that of non‐PABC when matching for stage, age, and histology,^9^, ^30^ most studies have reported that PABC is associated with a poorer prognosis.^5^, ^6^, ^7^, ^8^, ^31^ In our study, the tumor was aggressive with more patients at advanced T/N stage, and the five‐year OS and DFS rates were 72.3% and 56.1%, respectively, which indicated a poorer prognosis compared to non‐PABC patients. Recently, two studies showed still lower survival among PABC patients with less aggressive tumors (node negative, ER/PR positive), thus this aspect warrants further research.^32^, ^33^

There were some limitations to our study. First, this was a single center study with a limited population. Second, as the data was collected over a long period, there may be inconsistencies in the treatment methods. These factors may lead to relatively large discrepancies in the final results.

In conclusion, our findings confirm the adverse characteristics of PABC (advanced T/N stage, high rate of ER/PR negative, and HER2‐positive) and its poor prognosis. Ultrasound and mammography should be performed in PABC patients to avoid a delayed diagnosis. Diagnosis at earlier T/N stage and treatment with trastuzumab targeted therapy are beneficial to the prognosis of HER2‐positive PABC patients.

## Disclosure

No authors report any conflict of interest.
